# Supplementary material for: Development of automated whole breast 3D planning with tangential fields
Source: J Appl Clin Med Phys. 2025 Dec 10;26(12):e70415. doi: 10.1002/acm2.70415 (PMC12695690; doi:10.1002/acm2.70415)
Supplement: Supplementary file 1 — Supporting information [file ACM2-26-e70415-s001.docx]

**Development of Automated Whole Breast 3D Planning with Tangential Fields**

Christopher Busch^1^, Ping Xia^1,2^, Chirag Shah^4^, Rahul Tendulkar^1,2^, Young-Bin Cho^1,2,3^

^1^ Department of Radiation Oncology, Taussig Cancer Institute, Cleveland Clinic, 9500 Euclid Ave/CA-50, Cleveland, OH, 44195

^2^ Department of Radiation Oncology, Cleveland Clinic Lerner College of Medicine of Case Western Reserve University, 9501 Euclid Ave, Cleveland, OH, 44195

^3^ Department of Biomedical Engineering, Cleveland Clinic Lerner College of Medicine of Case Western Reserve University, 9501 Euclid Ave, Cleveland, OH, 44195

^4^ Department of Radiation Oncology, Allegheny Health Network, Pittsburgh, PA, 15222

**Running Title:** Automated Breast Planning

Corresponds to

Dr. Young-Bin Cho

Department of Radiation Oncology

Taussig Cancer Institute, Cleveland Clinic

9500 Euclid Ave / CA-50

Cleveland, Ohio, 44195

Choy2@ccf.org

**Development of Automated Whole Breast 3D Planning with Tangential Fields**

**Abstract:**

**Objectives/Purposes:** Whole breast 3D treatment planning with tangential fields often requires multiple manual adjustments, leading to inefficiency and variability. This study presents and evaluates an in-house developed automated script designed to improve planning efficiency and consistency for breast radiotherapy using tangential fields.

**Materials/Methods:** 19 breast cancer patients from an IRB-approved study were selected. The automated script was developed to generate beam segments through a three-step process, 1) verification of field setup, and generation of breast PTV, 2) duplication of the clinical plan with open tangents and 3) automatic generation of beam segments to reduce hotspot iteratively. Plans produced by the automated script (AS) were compared to the manually optimized (MO) clinical plans using eight evaluation metrics: V16Gy of the ipsilateral lung, mean heart dose, V90%, V95% (dose coverage), V105% (hotspot) of PTV, number of segments, MU (monitor unit) weighted segment area, and total MUs. A statistical analysis was performed using the Wilcoxon signed-rank test.

**Results:** Four of the eight metrics showed statistical differences. For MO and AS plans, the average ± SD of ipsilateral lung 16Gy was 15.05% ± 3.27% vs. 15.10% ± 3.26% (p=0.04) while the mean heart dose was 0.87 Gy ± 0.52 Gy, vs. 0.87 Gy ± 0.53 Gy (p=0.68). With regards to homogeneity and coverage, the PTV V105% were 2.0% ± 5.0% and 3.7% ± 6.4% (p=0.03) while the PTV V90% were 98.8% ± 1.5% and 99.1% ± 0.9% (p=0.14), V95% were 92.0% ±4.4% and 92.4% ± 3.2% (p=0.63) respectively. The number of segments and MU weighted segment areas were 7.3 ± 1.3 and 10.9 ± 1.9 (p<0.001); 268.2 ± 96.6 cm^2^ and 262.6 ± 92.9 cm^2^(p<0.001). The total MUs was 351.7 ± 99.0 and 354.7 ± 101.2 (p=0.05).

**Conclusion:** The automated script successfully generated high-quality tangential breast plans that met all clinical constraints. It significantly improved consistency while maintaining clinical acceptability, demonstrating potential for broader use in automated 3D planning.

**Keywords**: Automated Planning Script, Whole Breast Irradiation, Tangential breast planning, Plan Automation, Automated Segment Generation, Automated Forward Planning

1. **Introduction:**

Breast cancer is the leading cause of cancer related mortality among women, and the most frequently diagnosed life threatening cancer in female patients ^1^. As a result, it is one of the most common cancers encountered in oncology clinics ^2^. Standard treatment modalities for breast cancer include surgical options such as lumpectomy and mastectomy ^3^, radiation therapy (typically 45-50 Gy in 25 fractions) ^4^, and chemotherapy regimens ^5^. Radiation therapy is often the final step in a comprehensive treatment plan that may also involve surgery and/or chemotherapy ^6^.This paper focuses on whole breast tangential radiation therapy as an adjuvant therapy after lumpectomy surgery.

In breast treatment planning, the placement of tangential fields and the selection of beam energy depend on the patient’s body habitus, including considerations of appropriately shaping fields to protect critical organs such as the ipsilateral lung and heart. Modulation of tangential fields is typically achieved by adding a set of small segments within the open tangential fields (so called field-in-field (FIF) technique) without inverse planning. Small segments in FIF technique refer to the smaller, customized radiation fields created by the multi-leaf collimator (MLC). While the main field irradiates the entire target volume, these MLC-shaped segments target specific high-dose areas (or "hotspots"), delivering a reduced dose to them. The entire process of breast planning with two tangent fields can be tedious and time consuming. Due to the considerable variations in breast sizes among patients, significant differences in plan quality and planning efficiency are often observed in clinical practice. These challenges are not unique to tangential breast technique but are common to all 3D techniques involving multiple segments. In response, several centers have worked to automate 3D forward planning workflow for various anatomical sites ^7^. For example, a study from Kisling et al. implemented automated planning to generate four field box plans for cervical cancer based on anatomical landmarks ^7^.

The primary goal of this study is to develop an automated script (AS) capable of producing treatment plans with dosimetric quality equivalent to that of the manually optimized (MO) clinical plans for whole breast irradiation using tangential fields. The script was designed to mimic a clinical planning workflow used by experienced dosimetrists at our institution while minimizing the need for user intervention. The automated clinical workflow consists of three key steps; 1) Verifying prerequisites and requirements for appropriate open tangential fields (legitimacy test), and generating breast PTV once the requirements are met, 2) Creating a copy of the clinical plan with open tangential fields from step 1 and generating the necessary planning volumes required for segment generation in step 3 and 3) automatically generating segments and optimizing their weights. The script is modularized into three steps to allow dosimetrists to intervene if adjustments are needed. Plans generated with the automated script (AS) were compared to manually optimized (MO) clinical plans using several dosimetric and deliverability endpoints.

1. **Materials and Methods:**

Nineteen patients with breast cancers treated with whole breast radiation therapy were selected from a database approved by the institutional review board (CCF IRB 16-011). The treatment regimen includes one of two hypo fractionated prescriptions: 40.05 Gy in 15 fractions, or 26 Gy in 5 fractions (total three cases with one instance at 25 Gy in 5 fractions). The clinical plans were manually optimized (MO) by experienced dosimetrists at our institution using a forward planning field-in-field technique. In this approach, additional segment fields with smaller beam apertures were added to the open tangential fields to reduce the maximum dose within the breast tissue, ensuring compliance with the institutional dose guidelines. For this study, the clinical MO plans were retrospectively replanned using the in-house automated scripts (AS) implemented within the Pinnacle treatment planning system (Philips Medical Systems, Cleveland OH). The AS workflow enabled automated generation of optimized segments with a minimum user intervention. The resulting AS plans were then compared to the corresponding MO plans based in terms of plan quality and plan deliverability metrics.

The primary quality metric used for plan evaluation was PTV dose coverage, with a goal that the PTV receives at least 90% (or 95%) of the prescription dose, V90%, or V95%^8^.The secondary quality metric was the hotspot within the PTV, defined as volume of receiving 105% of prescription dose (V105%) as this may correlate with toxicity^8^. The two primary OAR dose constraints were the mean heart dose and the ipsilateral lung volume receiving 16 Gy (V16Gy). While the 5-fraction protocol's clinical constraint is typically V8Gy^9^ and the 15-fraction protocol's is V16Gy^10^. V16Gy was selected as a quality metric and V8Gy for 5fx protocol (only three patients) was not reviewed due to the limited number of cases. Mean heart dose is associated to cardiac toxicity which can lead to long term complications ^11, 12^, while lung toxicity is monitored through V16Gy, a metric primarily linked to the risk of pneumonitis ^13^ and secondary lung cancer ^12^. In the same token, 16 cases of 15fx protocol were analyzed for heart dose. In addition to dosimetric quality, plan deliverability was evaluated using three metrics: the number of segments, the monitor unit (MU) weighted segment area (defined in eq. 1), and total MUs. MU weighted segment area was specifically used to evaluate the degree of dose modulation with the generated segments.

| $MU weighted Segment Area=\Sigma(\frac{Mu per segment}{total MU}\times area of segment)$ | (1) |
| --- | --- |

**2.1 Manual Optimization**

Treatment field borders were outlined by radiopaque wires placed on the superior, inferior, medial and lateral parts of the breast by the attending physician prior to CT simulation. During simulation, patients were immobilized using a breast board, with/without the Active Breathing Coordinator (ABC) technique. For treatment planning, the wire markers were overridden with a density of 1g/cm^3^ to aid visualization without affecting dose computation. The two tangential breast fields were then placed by the dosimetrist with a collimator angle aligned parallel to the contour of the ipsilateral lung. The posterior border of fields was set inside of lung by 2-3 cm to account for setup uncertainty (a technique referred to a “lung bite”^14^) as a form of lung block. Initial dose calculations using these open tangential fields typically revealed a higher dose (hot spot) in the anterior portion of the breast. To mitigate these hotspots, regions exceeding 97-98% of the maximum dose were identified, and segment fields were created using multi-leaf collimators to block these areas. Each segment field is carefully weighted to reduce the overall hotspot. This forward planning process was repeated iteratively, with each additional segment typically reducing the hotspot dose by 2-3%, until the plan achieved adequate coverage (V100% ≥ 98%) and limited the maximum dose to below 107%, preferably ≤105%. The planning process also respected institutional constraints for critical organs, ensuring the ipsilateral lung V16Gy remained below 20%, and the mean heart dose was kept under 4Gy.

**2.2 Automated script (AS)**

An in-house script was developed within the Pinnacle TPS to automate labor intensive forward planning processes described in the previous section. The script assumes that key clinical steps such as a defined pair of tangential fields, beam energy and prescription are already in place during/after CT simulation. These are treated as “prerequisite conditions” for script execution. The automated script is organized into 3 main steps, steps as shown on the user interface in Figure 1. The first step, titled “Open Field Check” inspects and verifies whether all prerequisites are satisfied. The prerequisites include: the existence of tangential fields, an active prescription, availability of lung and heart contours, validity (“legitimacy’) of tangential fields, appropriate field sizes, proper lung blocks, and completion of open field dose computation. The legitimacy of tangential beam angles is determined by confirming that the pair of beams are in opposite quadrants of the coordinate space and their angular separation is within 180° ± 20°. Beam angle identified in quadrant space correctly distinguishes each Jaw direction, anterior or posterior to the breast. This check also distinguishes tangential fields pairs from anterior supraclavicular (AS) and posterior axillary (PA) beams, commonly included in 4 field breast plans to treat regional lymph nodes. Proper identification ensures that the script functions correctly whether used for two field tangential plans or more complex four-field configuration. In addition, the script checks the “pair-ness” of the beams, expecting one pair of legitimate tangential fields for single energy plan and multiple pairs for mixed energy tangential plans. When the default 10cm x 10cm beam or undefined block is used, the script alerts the user to verify the field size and lung block by displaying beam’s eye view. If any prerequisite is unmet, the script notifies the dosimetrist, allowing for correction before proceeding or completion of the missing task. Once all pre-requisites are satisfied, the script proceeds to automatically generate the planning target volume (PTV) of breast. This is done by computing 95% iso-dose line based on the dose distribution from paired open tangential fields. The resulting PTV generated by AS is shown in Figure 1. The location of dose calculation point, and normalization percentage in the prescription may be adjusted, allowing redefinition of the PTV if desired. The PTV can be replaced with a physician drawn PTV or the physician may manually edit the PTV before the second step. Since the fields are unmodulated at this point, significant hotspots may be present.

The second script, “Copy Field and Rx”, preserves the original tangential fields and prescription while creating a working copy of the plan for further processing. This step ensures that dosimetrists can revert to the original tangential fields at any time if needed. During this step dosimetrists may choose to experiment with various treatment techniques such as modifying beam energy or adding an additional pair of fields for mixed beam techniques. They may also activate dose contributions from AS/PA fields to ensure that the composite dose is accurately reflected especially at the junction regions for 4 field breast plans. Once the plan is copied, the script, “Copy Field and Rx” identifies anatomical sub regions of the breast specifically the anterior (“tip”) and posterior (“base”) portion of the PTV to assist in shaping the segment fields. The “tip” is defined by contracting the most posterior border of the tangential fields by 3 cm. The dose is then recalculated using this contracted field. The 95% isodose line from this recalculated dose distribution is used to define the “***tip***” of the breast, while the remaining portion of the PTV is designated as the “**base**.” This division of the breast into “***tip***” and “***base***” volumes enables the generation of optimally shaped segment fields using MLCs tailored to the anatomy and dose distribution. This process is also illustrated in Figure 1.

In the third step, the script iteratively identifies hotspots within the breast PTV, generates a segment field to block these hotspots and assigns appropriate weights to each segment. This process continues until either a pre-determined number of iterations (Eq. 2) or no hotspot (>105%) remains. The volume of hotspot is identified using a dynamic threshold starting at 97.5% of the current plan’s maximum dose initially. With each iteration, the script aims to reduce the hotspot intensity by approximately 2.5% assigning the segment a weight of 5% in the field (double the 2.5% hotspot). As the target dose becomes more homogeneous, both the hotspot threshold and the segment weight are dynamically reduced (see step 3 in Fig 1). Using volumetric Boolean operation, the identified hotspot volume is divided into two regions: the ***base*** and the ***tip*** of the breast. To address the hotspots in the ***base*** of the breast, the hotspot contour is extended posteriorly. This extended volume is used to generate a ‘blocked aperture’. For the hotspots in the tip, the volume is extended anteriorly to generate the appropriate shape of ‘blocked aperture’. These extensions ensure correct aperture design for the segment, which will be subsequently converted into MLC; without these volumetric operations, segment creation may fail or result in suboptimal MLC configuration. Once aperture design for the segment is completed, this blocked field is converted with MLC segment field using the function of Pinnacle TPS. At this stage, appropriate weighting (e.g., 5% weight to reduce a 2.5% hotspot when the threshold of 97.5% was used) was applied to balance dose coverage and hotspot suppression. This process is repeated sequentially for each beam and cycles continue until the maximum number of allowed segments is reached (Eq 2) or no remaining hotspot is detected. The process of segment generation is illustrated in Figure 2. Figure 2A visualizes the hotspot as a contour. This contour is then extended and used to generate blocks as shown in Figure 2B. Figure 3 shows an example of the sequence of generated segments. The entire automated process is depicted in Figure 1.

| $Maximum number of segments=int(\frac{Dmax-105\%}{2.5\%})$ | (2) |
| --- | --- |

Where Dmax is the max dose in % of the prescription dose.

After all the segments are generated, segment weight optimization is performed to further improve dose homogeneity within the breast PTV. Minimum MU per segment was limited to 4, while uniform dose and maximum dose constraint to PTV were both applied with a weight of 50 for segment weight optimization. All processes in the third script are executed automatically without requiring any dosimetrist intervention.

Most of the script's execution time occurs in step 3, which involves 3D volume operations for hotspot identification, segment field definition and repeated dose calculations. It scales with the number of segments created. The performance was measured using the patient case that generated the maximum number of segments (14). A Wilcoxon signed-rank test was performed for each metric using a significance level of 0.05 to determine statistical significance using SAS v9.4 (SAS Institute, Cary, NC).

1. **Results:**

To objectively evaluate the treatment plan performance of the MO and AS plans, eight metrics were assessed. These metrics included five dosimetric quality indicators; the ipsilateral lung V16Gy, mean heart dose, V90%, V95% (coverage) and V105% (hotspot) of PTV as shown in Figure 4, and three plan deliverability indicators; MU weighted open segment area, number of segments, and total MU as illustrated in Figure 5. The clinically used MO plans served as the baseline for acceptable plan quality, while the AS plans were compared against this standard. Table 1 summarizes the mean and standard deviation of each metric across the entire data set, comparing the MO and AS plans.

- 1. **Dosimetric Quality Metrics**

The first two planning metrics evaluated were OAR constraints. The ipsilateral lung V16Gy demonstrated a statistically significant difference between MO and AS plans (p = 0.04), as indicated by the asterisk in the boxplot of Figure 4A. Despite the statistical difference, the mean V16Gy value of lung were nearly identical; 15.05% for MO and 15.10% for AS plans. Both datasets included a single high outlier corresponding to the same patient. When the outlier is excluded, the statistical difference is no longer present. The second OAR metric assessed was the mean heart dose, shown on Figure 4B. No statistically significant difference was found between the two planning methods (p = 0.68). Both plan types exhibited a single high outlier, which occurred in a left breast case where part of the heart was within the treatment field. A more comprehensive lung block during planning may have reduced the mean heart dose for this patient. The mean heart dose for the MO dataset was 0.87 Gy ± 0.52 Gy, while the AS dataset yielded 0.87 Gy ± 0.53 Gy.

The next three metrics evaluated were related to the planning target volume (PTV). The V105%, representing the volume of the PTV receiving more than 105% of the prescription dose, reflects the optimizer’s ability to mitigate hotspots. Figure 4C shows a statistically significant difference between the MO and AS plans for this metric (p = 0.03). Multiple high outliers were noted, but unlike the previous metrics, they did not consistently appear on the same patients, suggesting no clear correlation. When one of the outliers is excluded, the statistical difference is no longer present as is the case of Lung V16Gy. The average V105% was 2.0% ± 5.0% for the MO plans and 3.7% ± 6.4% for the AS plans. The final dosimetric metric, PTV coverage (V90% and V95%), was defined as the percentage of the PTV receiving at least 90% (or 95%) of the prescription dose. The difference in V90% was not statistically significant (p = 0.14). Two outliers with reduced coverage in the MO dataset and one in the AS dataset were patients treated under special clinical circumstances where localized boost fields were used in conjunction with the tangential fields. As a result, evaluating coverage using only the tangential fields in this simulation may have underestimated the true target coverage in these cases. The average V90% was 98.8% ± 1.5% for the MO plans and 99.1% ± 0.9% for the AS plans. The V95% was found to be 92.0% ± 4.4% and 92.4% ± 3.2%, respectively. These results are depicted in Figure 4D and 4E.

- 1. **Deliverability Quality Metrics**

Among all eight metrics analyzed, the number of segments demonstrated the most statistically significant difference between MO and AS plans (p < 0.001), as shown in Figure 5A. The average number of segments for the MO plans was 7.3 ± 1.3 compared to 10.9 ± 1.9 for AS plans. Nearly all AS data points exceeded the upper quartile of the MO dataset, indicating that the AS plans generally used more segments to achieve dose homogeneity. Another statistically significant difference was observed when comparing MU weighted open segment area between the two planning approaches (p < 0.001). Based on Equation 1, the average MU-weighted open area for the MO plans was 268.2 ± 96.6 cm², while the AS plans yielded an average of 262.6 ± 92.9 cm². This comparison is visualized in Figure 5B. Outliers in both datasets were associated with the same patient case, likely to reflect difficulties maximizing PTV coverage during planning.

The final plan delivery metric evaluated was the total number of MUs. As shown in Figure 5C, there was no statistically significant difference in total MUs between the MO and AS plans (p = 0.05). The average total MU was 351.7 MUs ± 99.0 for the MO plans and 354.7 MUs ± 101.2 MUs for the AS plans. While the AS plans exhibited a slightly higher MU on average, the difference was small and well within clinical acceptability. These results suggest that despite using more segments, the AS plans did not significantly increase overall beam-on time or complexity in terms of total MU.

The execution time for the patient with the maximum segment number of 14 was 22s, 8s and 5 minutes 24s for each step of script.

| Table 1: Patient plan characteristic results. | | | |
| --- | --- | --- | --- |
| Plan Evaluation criteria | MO plans | AS plans | P value |
| *Ipsilateral lung V16Gy [%] | 15.05% ± 3.27% | 15.10% ± 3.26% | 0.04 |
| Mean Heart Dose [Gy] | 0.87 ± 0.52 | 0.87 ± 0.53 | 0.68 |
| *PTV V105% [%] | 2.0% ± 5.0% | 3.7% ± 6.4% | 0.03 |
| PTV coverage (V90%) [%] | 98.8% ± 1.5% | 99.1% ± 0.9% | 0.14 |
| PTV coverage (V95%) [%] | 92.0% ± 4.4% | 92.4% ± 3.2% | 0.63 |
| *Number of Segments | 7.3 ± 1.3 | 10.9 ± 1.9 | <0.001 |
| *MU weighted Segment Area (cm^2^) | 268.2 ± 96.6 | 262.6 ± 92.9 | <0.001 |
| Total Plan MU | 351.7 ± 99.0 | 354.7 ± 101.2 | 0.05 |

*** Indicates a statistically significant**

Averages ± standard deviation, comparing manual and automated plans.

16 patients in 15 fx protocol for Lung V16Gy and Heart dose.

1. **Discussion:**

This study demonstrates that the in-house automated script (AS) can generate forward-planned whole breast radiation therapy plans, achieving excellent dosimetric and deliverability metrics. The automated process closely mimics the established field-in-field technique used by experienced dosimetrists, while offering improved efficiency and reproducibility with minimal user input.

The PTV coverage (V90% and V95%) was well preserved in the AS plans, with no statistically significant difference compared to MO plans. Importantly, the AS plans also met critical organ-at-risk (OAR) constraints. Although the ipsilateral lung V16Gy was statistically different between AS and MO (p = 0.04), this difference was clinically insignificant at 15.05% vs 15.10%. When one outlier is excluded, the statistical difference is no longer present. The mean heart dose showed no statistically significant difference, affirming the script's ability to maintain heart sparing even in left-sided cases. Notably, small discrepancies may be further reduced with improved lung block contouring or additional script refinements. A statistically significant difference was observed in the V105% (hotspot volume), with AS plans showing slightly higher values than MO plans (3.7% vs. 2.0%, p = 0.03). However, the observed V105% values remained within clinical tolerances, indicating dosimetric acceptability of AS plans. As illustrated in Figure 4C, multiple data points fall outside of two standard deviations. When any one of the outliers was excluded from the analysis, the statistical difference for the V16Gy was no longer observed. We acknowledge that a larger sample size is needed to validate the stability of this result.

Deliverability analysis revealed that AS plans consistently generated more segments than MO plans (10.9 vs. 7.3, p < 0.001), reflecting a more systematic and iterative approach to hotspot reduction. Despite the increased number of segments, the total MUs were statistically insignificant between the two groups, suggesting that the script achieves plan homogeneity without compromising treatment efficiency. Additionally, while the MU-weighted open segment areas showed a statistically significant difference, the actual magnitude was minimal, supporting the script’s ability to closely replicate the open field geometry used in MO plans. A careful analysis of segment field size revealed that MO plans exhibited greater variability, with a peak distribution at around 20% of the corresponding open tangential field. In contrast, AS plans demonstrated a more consistent distribution in segment size, peaking at around 60% of the open tangential field (see supplement Figure 1).

Some centers have already incorporated automated planning into their routine clinical workflow^15^. However, challenges have been reported, including over-sparing of organs-at-risk (OARs)^16^ and overall suboptimal plan quality in certain cases^17^. One study attributed decreased plan quality to incorrect or overly simplistic input parameters^17^, underscoring the importance of proper configuration and validation. While successful implementation of automated forward planning has been demonstrated, caution is warranted before widespread clinical adoption without thorough site-specific testing and quality assurance^18^. The evaluation in this study focused on a single treatment site—whole breast irradiation using tangential fields—a relatively straightforward case for which commercial solutions exist^19^. Nonetheless, our findings support the feasibility of developing an in-house automated planning tool capable of meeting all clinical constraints and providing acceptable plan quality.

Although limited in scope to breast treatments, our results align with other successful applications of automated planning in different anatomical sites, such as whole brain radiotherapy^20^. Moreover, ongoing research is exploring the feasibility of automated planning in more complex treatment sites like head and neck cancers^21^, which often involve challenging geometry and stricter dose constraints. Importantly, automated planning systems have been successfully implemented across various treatment planning platforms^15, 20^, indicating broader industry recognition of automation as a promising direction for radiation oncology.

The algorithm of segment field generation demonstrated in this study can be readily generalized to other treatment sites. With minimal modification, the algorithm has been adapted to generate whole brain treatment plans. In this case, beam legitimacy test was revised to identify parallel opposed fields instead of opposite quadrant constraints used for breast tangents. Additionally, the posterior field (the tip structure of breast) was redefined from posterior border to isocenter position. This revised script is currently undergoing validation at our institution. The concept of segment creation in conformal 3D planning can be further extended in the same way. Although the current study focuses on the relatively simple tangential breast technique, the same script is capable of generating plans for breast treatments including regional lymph nodes by adding an anterior supraclavicular field (or a supraclavicular field plus a posterior axillary boost field) without additional modification. Notably the automated script significantly improves dose homogeneity at the junction region of the 4 field plans. Furthermore, the current version of the script can accommodate mixed energy tangential plans, demonstrating its flexibility and broad applicability in clinical practice.

The definition of hotspots in this study begins at 97.5% of the maximum dose (representing 2.5% hotspots), as the dose fall-off in dose volume histogram is relatively gradual. With each successive segment, the hotspot threshold is linearly reduced to 1% at the last segment, since in a more homogeneous dose distribution, even a 1% hotspot volume represents a clinically meaningful volume to shape the segment. The automated script uses a forward planning approach to generate segment fields, whereas dosimetrists often apply continuous modification of the existing segment (manual inverse planning) during the segment generation. As a result, manual optimization is typically time-consuming and labor-intensive. One limitation of the current forward planning script is that it cannot retrospectively modify previously generated segment fields. Consequently, some segments may appear suboptimal when viewed in the context of the complete inverse plan optimization.

Despite this limitation, the automated script consistently produces baseline plans of clinically acceptable quality. Moreover, as demonstrated in Figure 6, dosimetrists can achieve significant improvements with minimal manual adjustments—such as modifying a few MLC tips—which leverage inverse planning principles. It is important to note that the results presented in this study reflect unmodified plans produced solely by the automated script, without any post-processing or manual fine-tuning. This highlights the baseline capabilities of the AS-generated plans and the ease with which they can be enhanced if needed.

This study adds to the growing body of evidence that automated planning, when carefully developed and validated, can match the quality and safety of manual planning while improving efficiency and consistency. Overall, the AS demonstrates robust performance in creating high-quality, clinically deliverable treatment plans. Its modular structure allows user oversight and intervention if needed, making it a flexible tool for streamlining the planning workflow.

1. **Conclusion:**

An in-house developed automated tangential planning script for whole breast irradiation has demonstrated both robustness and efficiency. The script consistently generated high-quality treatment plans within approximately five minutes, meeting all clinical evaluation metrics typically used for manual planning. Overall, this automated planning approach offers a reliable and effective solution to streamline the treatment planning process, enhance workflow efficiency, and maintain high standards of plan quality and patient safety for 3D tangential breast radiotherapy.

| 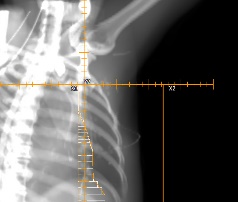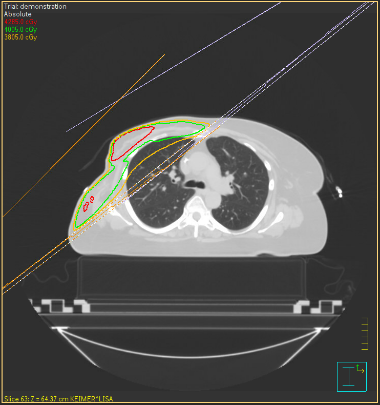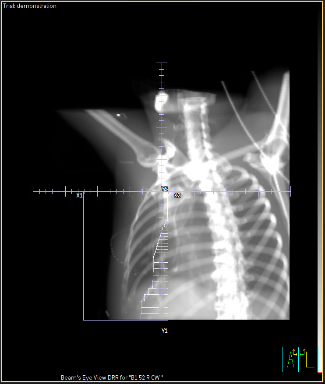 [Simulation]  Set tangential fields.  [Prescription]  Dose computation with open fields.  PTV = 95% Rx volume  **0. Prerequisites**  **0. Validity (“Legitimacy”) Check**  Existence of Lung/Heart contour,  Appropriate field size, Proper lung blocks,  ‘Pair-ness’ of tangential beams. 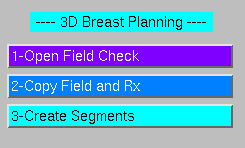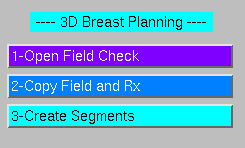 Check if all prerequires and Legitimacies are satisfied.  If these are unmet, stop proceeding, list the issues and allow dosimetrists correct them.  Generate PTV contours from 95% iso dose line to be reviewed and modified if necessary. Register all planning variables for the next step. 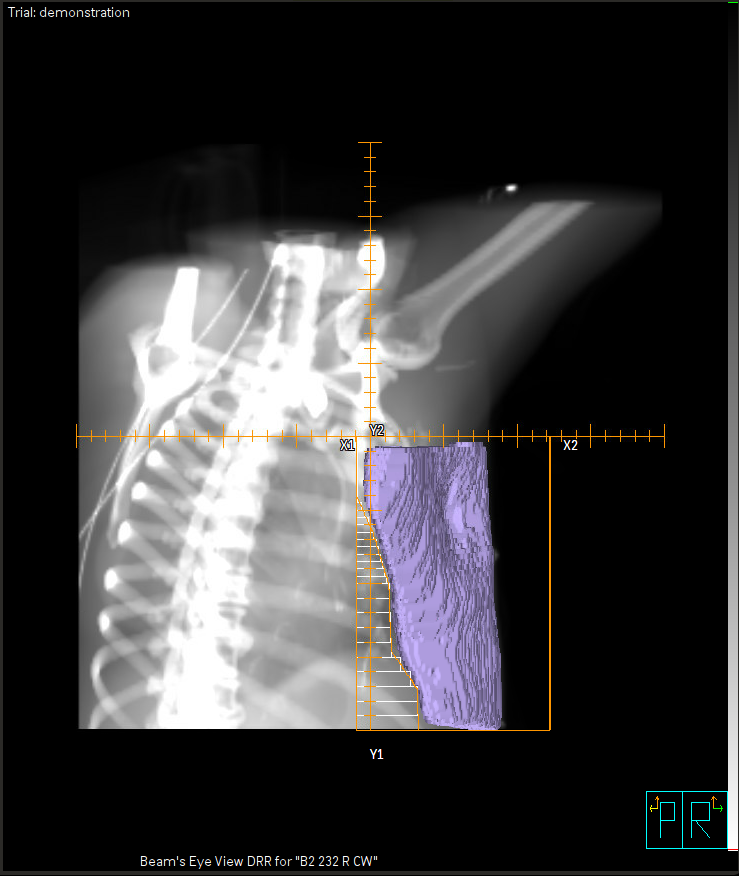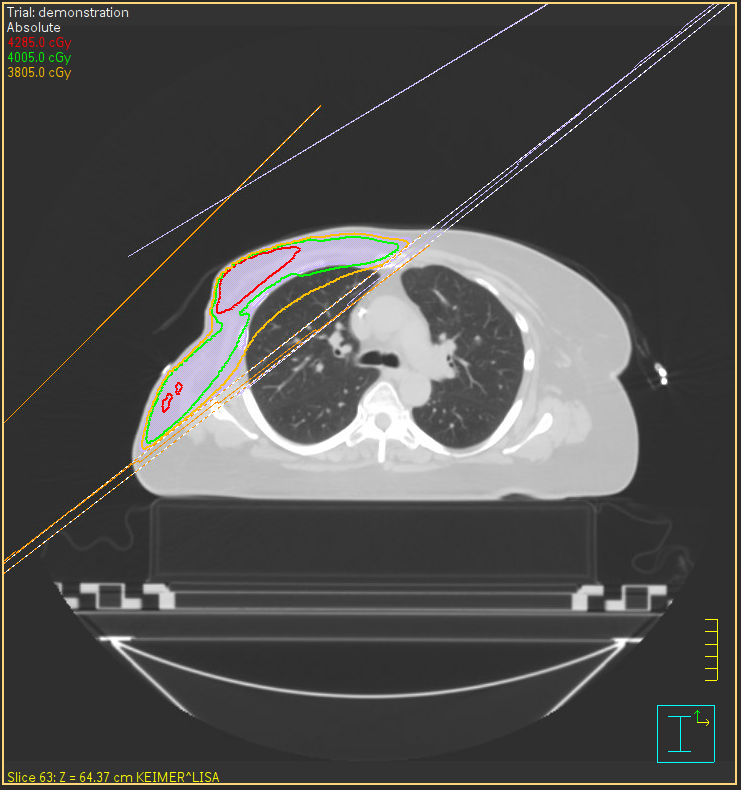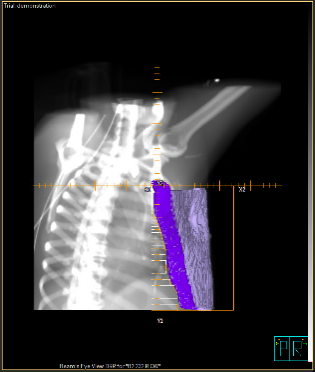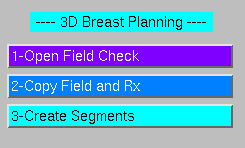 Determine the ***tip*** (lighter shade) and ***base*** (darker shade) portion of PTV. Hotspot within ***base*** will be blocked by posterior MLCs. Preserve the original beams and Rx and create a working copy of the plan. 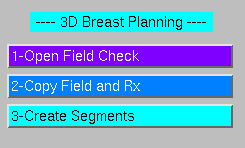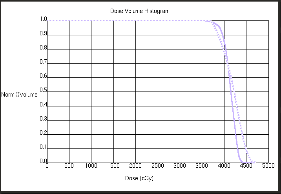 Volume of hotspot is determined by the threshold as a function of maximum dose (Left). As segments added, dose gradient gets sharper (right) and the threshold needs to be increased. 3D volume of hotspot is then divided into the base and tip part of PTV. 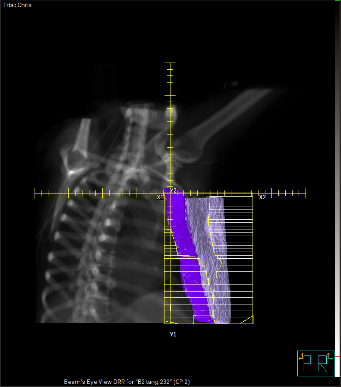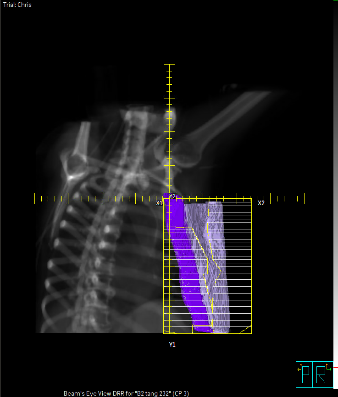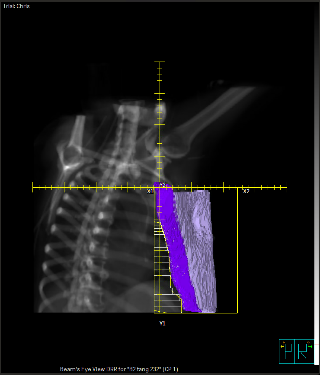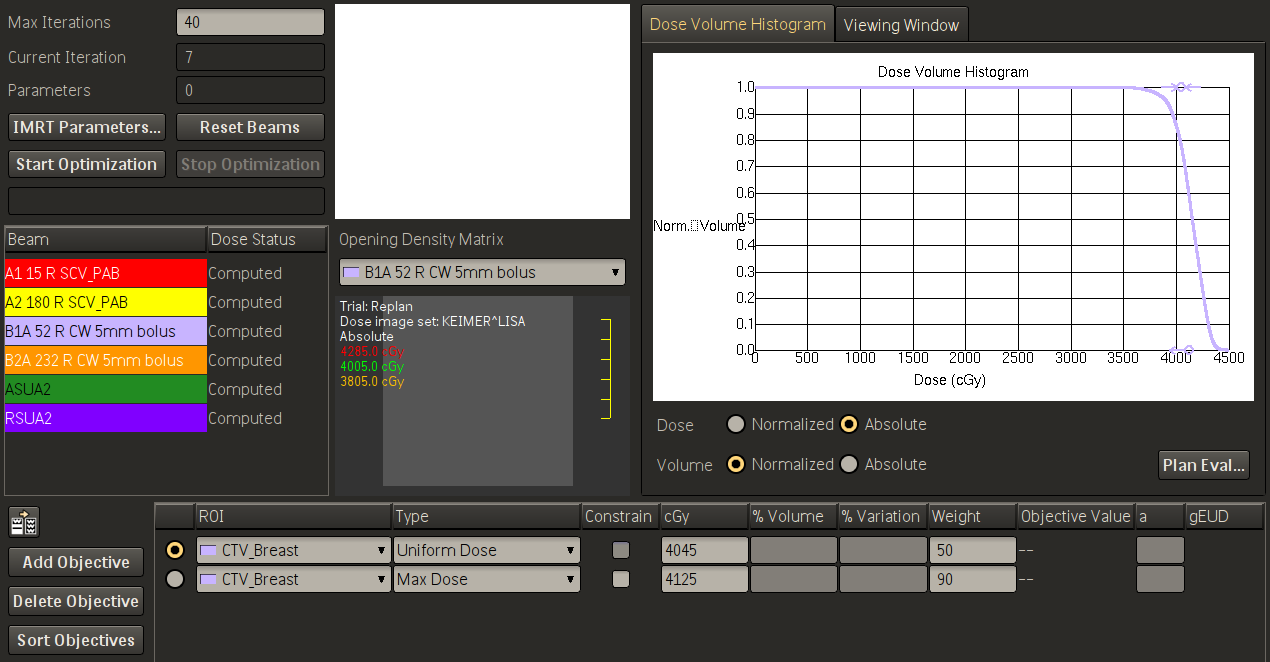 Segments are generated till the pre-defined number, int ((max dose -105)/2.5). Segment weights are optimized. |  |
| --- | --- |
|  | |
| Figure 1. Automated Script Planning Workflow. The planning workflows were implemented with three steps or three buttons in the user interface. Prerequisites and beam legitimacy are expected to be in place before running the script. The first script inspects and validates prerequisites and beam legitimacy. Once all met, breast PTV is automatically generated. The second script prepares a working copy of the plan and distinguishes the ***tip*** and ***base*** portion of the breast PTV. The third script automatically generates segment fields to optimize dose distribution within the breast PTV. | |

| 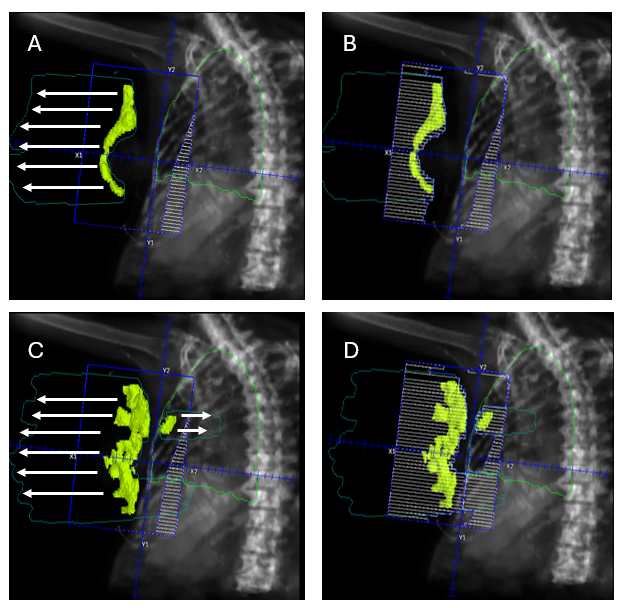 |
| --- |
| Figure 2. Segment field generation. Hotspots are initially identified from the dose distribution of the open tangential field pairs as shown in (A). In early iteration, hotspots typically appear in the anterior portion of the breast (***tip***) and are projected anteriorly to define the appropriate segment block. The blocked field is then converted with MLCs in (B). As the dose distribution becomes more homogeneous through successive segment addition, remaining hotspots tend to appear in both the tip and the base of the breast PTV. Hotspots in the base is projected posteriorly to define the field blocks for segment (C). Finally segment field is generated by converting the blocked segment field (D). |


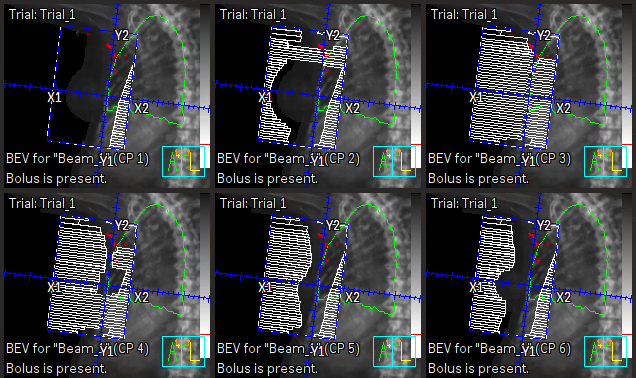


Figure 3. Generated Segments. The first control point (CP1, upper left corner) represents the open tangential field, and the remaining CPs are segment fields automatically generated by the script. Notably, the lung block defined by dosimetrist in CP1 is preserved throughout all segment fields. The final hotspot (99% of maximum dose shown in red volume) is located at the tip of MLCs in CP3.

Figure 4. Dosimetric plan quality metrics. (* indicates statistical significance). (A) Ipsilateral lung V16Gy shows a statistically significant difference (p = 0.04); mean of 15.05% for the manually optimized (MO) vs. 15.10% for the automated script (AS) plans. (B) Mean heart dose shows no statistically significant difference between MO and AS plans (p = 0.68). (C) Hotspot volume (V105%) is statistically significant (p = 0.03): 2.0% for MO vs. 3.7% for AS plans. As will be discussed in Fig 6, it can be easily remedied. (D) PTV coverage (V90%) shows no statistically significant difference (p = 0.14), with both techniques achieving high coverage. The PTV V95% also did not exhibit a statistically significant difference. All outliers in (C) satisfied the institutional V107% constraints (V107%<3%).

Figure 5. Plan deliverability metrics (* indicates statistical significance). (A) Number of segments for manually optimized (MO) and automated script (AS) plans shows a statistically significant difference. MU-weighted segment area (cm²) also showed a statistically significant difference (B), while the total MU did not (C).

**
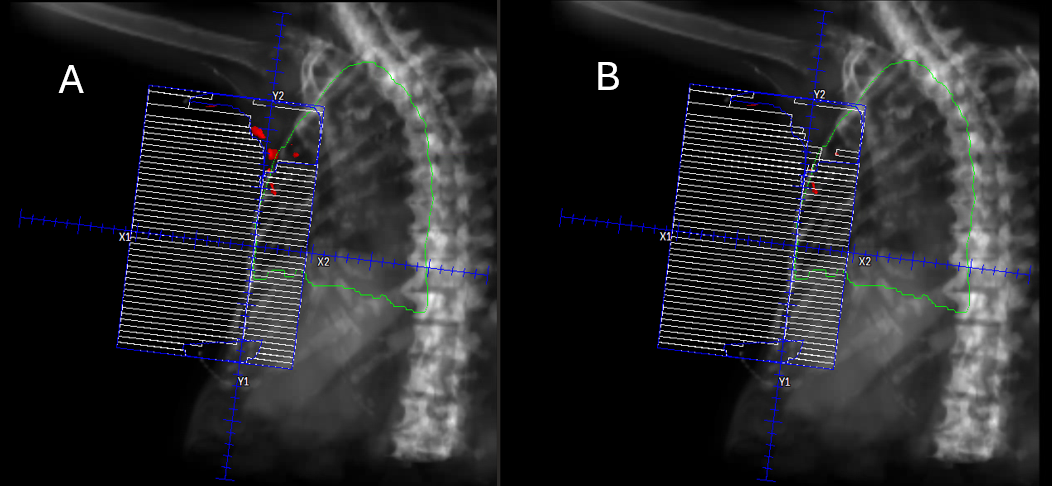
**

Figure 6: Final plan adjustment. The remaining hotspots (99% of maximum dose in this example) are often localized at the tip of MLCs in one of the control points as shown in (A), reproduced from CP3 in Figure 3. Dosimetrists can perform a final adjustment by manually modifying the position of a few MLC tips, which frequently results in a significant reduction in the hotspot volume with minimum intervention (B).

**Supplement Fig 1:** Histogram of segment area between AS and MO datasets

**Author Contributions**

Conception and design: Ping Xia, Young-Bin Cho; Script development: Young-Bin Cho; Data collection: Christoper Busch; Data analysis and interpretation: Christoper Busch, Young-Bin Cho; Manuscript Writing: Christoper Busch, Ping Xia, Chirag Shah, Young-Bin Cho; Final approval of manuscript: Rahul Tendulkar, Young-Bin Cho**.**

**Conflict of Interest**

The authors have no relevant conflicts of interest to disclose.

**Acknowledgements**

We would like to thank the radiation dosimetrists in our institution for their vital contributions to script validation. Their expertise and meticulous feedback improve the quality of these scripts.

**Ethical approval statement**: Nineteen patients with breast cancers treated with whole breast radiation therapy were selected from a database approved by the institutional review board (CCF IRB 16-011).

**References**

1. Sharma GN, Dave R, Sanadya J, Sharma P, Sharma K. Various types and management of breast cancer: an overview. *Journal of advanced pharmaceutical technology & research*. 2010;1(2):109-126.

2. McPherson K, Steel C, Dixon J. ABC of breast diseases: breast cancer—epidemiology, risk factors, and genetics. *BMJ: British Medical Journal*. 2000;321(7261):624.

3. Riis M. Modern surgical treatment of breast cancer. *Annals of medicine and surgery*. 2020;56:95-107.

4. CENTER NO, CENTER NB, HEADQUARTERS R, CENTER RS. NSABP PROTOCOL B-39 RTOG PROTOCOL 0413.

5. Anampa J, Makower D, Sparano JA. Progress in adjuvant chemotherapy for breast cancer: an overview. *BMC medicine*. 2015;13:1-13.

6. Sas-Korczyńska B, Śladowska A, Rozwadowska-Bogusz B, et al. Comparison between intensity modulated radiotherapy (IMRT) and 3D tangential beams technique used in patients with early-stage breast cancer who received breast-conserving therapy. *Reports of Practical Oncology & Radiotherapy*. 2010;15(4):79-86.

7. Kisling K, Zhang L, Simonds H, et al. Fully automatic treatment planning for external-beam radiation therapy of locally advanced cervical cancer: a tool for low-resource clinics. *Journal of global oncology*. 2019;5:1-9.

8. Smith BD, Bellon JR, Blitzblau R, et al. Radiation therapy for the whole breast: Executive summary of an American Society for Radiation Oncology (ASTRO) evidence-based guideline. *Practical radiation oncology*. 2018;8(3):145-152.

9. Brunt AM, Haviland JS, Wheatley DA, et al. Hypofractionated breast radiotherapy for 1 week versus 3 weeks (FAST-Forward): 5-year efficacy and late normal tissue effects results from a multicentre, non-inferiority, randomised, phase 3 trial. *The Lancet*. 2020;395(10237):1613-1626.

10. Vicini F, Winter K, Freedman G, et al. NRG RTOG 1005: a phase III trial of hypo fractionated whole breast irradiation with concurrent boost vs. conventional whole breast irradiation plus sequential boost following lumpectomy for high risk early-stage breast cancer. *International Journal of Radiation Oncology, Biology, Physics*. 2022;114(3):S1.

11. Darby SC, Ewertz M, McGale P, et al. Risk of ischemic heart disease in women after radiotherapy for breast cancer. *New England Journal of Medicine*. 2013;368(11):987-998.

12. Brownlee Z, Garg R, Listo M, Zavitsanos P, Wazer DE, Huber KE. Late complications of radiation therapy for breast cancer: evolution in techniques and risk over time. *Gland surgery*. 2018;7(4):371.

13. Goldman UB, Wennberg B, Svane G, Bylund H, Lind P. Reduction of radiation pneumonitis by V20-constraints in breast cancer. *Radiation oncology*. 2010;5:1-6.

14. Xia P, Godley A, Shah C, Videtic GM, Suh J. *Strategies for radiation therapy treatment planning*. Springer Publishing Company; 2018.

15. Gleeson I, Bolger N, Chun H, et al. Implementation of automated personalised breast radiotherapy planning techniques with scripting in Raystation. *The British Journal of Radiology*. 2023;96(1144):20220707.

16. Moore KL. Automated radiotherapy treatment planning. Elsevier; 2019:209-218.

17. Wortel G, Eekhout D, Lamers E, et al. Characterization of automatic treatment planning approaches in radiotherapy. *Physics and Imaging in Radiation Oncology*. 2021;19:60-65.

18. Wang M, Zhang Q, Lam S, Cai J, Yang R. A review on application of deep learning algorithms in external beam radiotherapy automated treatment planning. *Frontiers in oncology*. 2020;10:580919.

19. Dragojević I, Hoisak JD, Mansy GJ, Rahn DA, Manger RP. Assessing the performance of an automated breast treatment planning software. *Journal of Applied Clinical Medical Physics*. 2021;22(4):115-120.

20. Han EY, Cardenas CE, Nguyen C, et al. Clinical implementation of automated treatment planning for whole‐brain radiotherapy. *Journal of applied clinical medical physics*. 2021;22(9):94-102.

21. McIntosh C, Welch M, McNiven A, Jaffray DA, Purdie TG. Fully automated treatment planning for head and neck radiotherapy using a voxel-based dose prediction and dose mimicking method. *Physics in Medicine & Biology*. 2017;62(15):5926.
